# Positive association between plasmatic levels of orexin A and the endocannabinoid-derived 2-arachidonoyl lysophosphatidic acid in Alzheimer’s disease

**DOI:** 10.3389/fnagi.2022.1004002

**Published:** 2022-11-16

**Authors:** Nicola Forte, Alba Clara Fernández-Rilo, Letizia Palomba, Brenda Marfella, Fabiana Piscitelli, Paolo De Girolamo, Alfonso Di Costanzo, Vincenzo Di Marzo, Luigia Cristino

**Affiliations:** ^1^Institute of Biomolecular Chemistry, Consiglio Nazionale delle Ricerche, Pozzuoli, Italy; ^2^Department of Biomolecular Sciences, University of Urbino Carlo Bo, Urbino, Italy; ^3^Department of Veterinary Medicine and Animal Productions, University Federico II, Naples, Italy; ^4^Department of Medicine and Health Sciences Vincenzo Tiberio, Centre for Research and Training in Medicine of Aging, University of Molise, Campobasso, Italy; ^5^Heart and Lung Research Institute, Université Laval, Québec City, QC, Canada; ^6^Institute for Nutrition and Functional Foods, Université Laval, Québec City, QC, Canada; ^7^Centre NUTRISS, Université Laval, Québec City, QC, Canada

**Keywords:** hypocretin (orexin), sleep, neurodegeneration, tau phosphorylation, serum biomarker

## Abstract

A regular sleep-wake cycle plays a positive function that preserves synaptic plasticity and brain activity from neuropathological injuries. The hypothalamic neuropeptide orexin-A (OX-A) is central in sleep-wake regulation and has been found to be over-expressed in the cerebrospinal fluid (CSF) of patients with Alzheimer’s disease (AD) suffering from sleep disturbances. OX-A promotes the biosynthesis of 2-arachidonoylglycerol (2-AG), which, in turn, could be phosphorylated to 2-arachidonoyl lysophosphatidic acid (2-AGP). The reorganization of the actin cytoskeleton during neurite retraction is one of the best-characterized effects of lysophosphatidic acids. However, less information is available regarding the reorganization of the neuronal microtubule network in response to OX-A-induced 2-AG and, possibly consequent, 2-AGP production in AD patients. This is of special relevance also considering that higher 2-AG levels are reported in the CSF of AD patients. Here, we found a positive correlation between OX-A and 2-AGP concentrations in the plasma, and an increase of 2-AGP levels in the CSF of AD patients. Furthermore, a negative correlation between the plasmatic 2-AGP levels and the mini-mental state examination score is also revealed in AD patients. By moving from the human patients to *in vitro* and *in vivo* models of AD we investigated the molecular pathway linking OX-A, 2-AG and 2-AGP to the phosphorylation of pT231-Tau, which is a specific early plasma biomarker of this disorder. By LC-MS analysis we show that OX-A, via OX-1R, induces 2-AG biosynthesis via DAGLα, and in turn 2-AG is converted to 2-AGP in primary hippocampal neurons. By confocal microscopy and western blotting assay we found an OX-A- or 2-AGP-mediated phosphorylation of Tau at threonine 231 residue, in a manner prevented by LPA1R (2-AGP receptor) or OX1R (OX-A receptor) antagonism with AM095 or SB334867, respectively. Finally, by patch-clamp recording we documented that 2-AGP-mediated pT231-Tau phosphorylation impairs glutamatergic transmission in the mouse hippocampus. Although further additional research is still required to clarify the potential role of orexin signaling in neurodegeneration, this study provides evidence that counteraction of aberrant OX-A signaling, also via LPA-1R antagonism, may be beneficial in the mild-to-moderate age-related cognitive decline associated with sleep disturbances.

## Graphical Abstract

Schematic representation of sleep-wake alteration cycle on neuronal plasticity of orexin system and its putative effect on neuronal degeneration induced by enhancement of OX-A, 2-AG and 2-AGP production. **(A)** Sleep disturbances and insomnia trigger orexin neuron excitability which, in turn, further promote the wake and arousal state together with the production of 2-AGP whose levels have been found positively correlated to those of OX-A in the plasma of AD patients. **(B)** OX-A via OX-1R induces 2-AG biosynthesis through activation of phospholipase C (PLC). 2-AG is then phosphorylated to 2-AGP via MAG Kinase enzymatic activity. **(C)** Schematic representation of microtubule disassembly triggered by pT231-Tau phosphorylation downstream to 2-AGP signaling at LPA1R activation. 
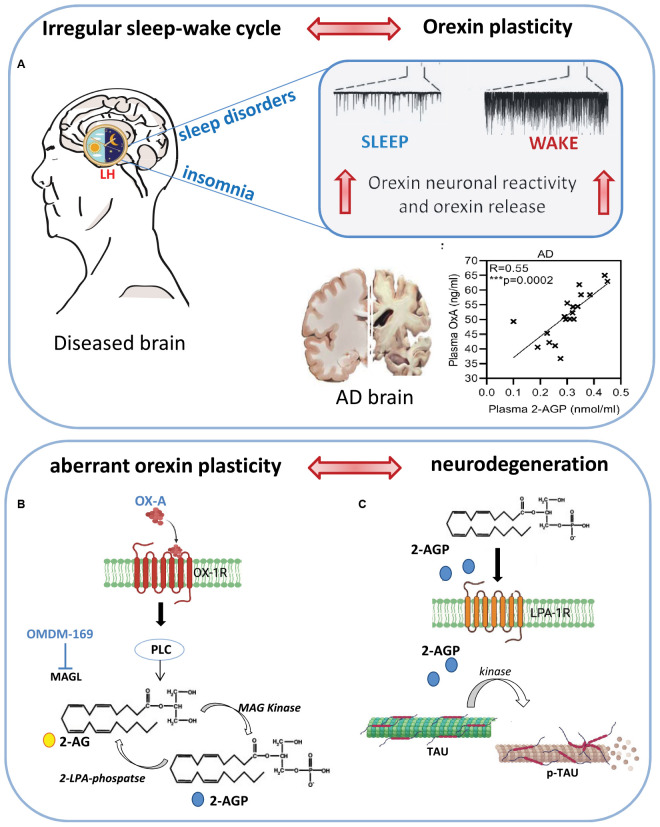

## Introduction

Alzheimer’s disease disorder (AD) is the most common cause of dementia characterized by progressive and fatal neurodegeneration, deterioration of memory and cognitive functions, progressive inability to perform normal activities of daily life, neuro-psychiatric symptoms, and behavioral alterations (McKhann et al., 2011).

Progressive cognitive and behavioral impairments in AD are frequently associated with the deregulation of the sleep-wake cycle. Indeed, cerebrospinal fluid (CSF) biomarkers such as Aβ42 and Aβ40, t-tau and p-tau significantly correlate with the reduction of nocturnal sleep (Liguori et al., 2021). A regular sleep-wake cycle plays a positive function that preserves synaptic plasticity and brain activity from the neuropathological brain injuries triggered by neurofibrillary degeneration and amyloid-β (Aβ) plaque accumulation (Tononi and Cirelli, 2006; Mander et al., 2016) in the parenchyma and CSF of cognitively healthy older subjects (Ju et al., 2013; Spira et al., 2013; Sprecher et al., 2015). Accordingly, epidemiological data report sleep disturbances in about half of AD patients (Liguori et al., 2021).

In line with these data, deregulation of the orexinergic system has been documented in AD patients, who show increases in orexin-A (OX-A) levels in the CSF (Osorio et al., 2016; Gabelle et al., 2017; Shimizu et al., 2020), and in AD animal models (Liu et al., 2018; Zhao et al., 2022). OX-A (or hypocretin 1) is a 33-amino acid peptide produced by a small number of neurons strictly localized in the lateral hypothalamic area (Peyron et al., 1998), which send projections widely distributed throughout the brain (Peyron et al., 1998; Date et al., 1999; Nambu et al., 1999). Although OX-A is involved in several homeostatic functions including feeding behavior, energy homeostasis, stress response and the reward, its role in sleep-wake regulation is central by stabilizing transitions between sleep and wake mostly through innervations of wake-promoting neurons in the central nervous system (de Lecea, 2021).

Of note, subjects suffering of type 1 narcolepsy, who have low levels of orexins in the CSF, also present lower CSF amyloid-b (Ab) and phosphorylated (p-tau) levels as compared to unaffected healthy controls (Jennum et al., 2017). Moreover, OX-A plays also an important role in learning and memory mainly by modulating hippocampal-dependent cognitive tasks (Shimizu et al., 2020; Forte et al., 2021). OX-A is a highly lipophilic neuropeptide that has been detected in both CSF and plasma, and it is able to cross the blood-brain barrier in both directions (Kastin and Akerstrom, 1999). OX-A shows the highest affinity for the orexin receptor 1 (OX-1R), a G-protein-coupled receptor (GPCR) preferentially coupled to Gq that has been found to modulate endocannabinoid-mediated synaptic plasticity via phospholipase C (PLC), phospholipase A2 (PLA2), and phospholipase D (PLD) signaling (Kukkonen and Leonard, 2014). In particular, PLC activation triggers diacylglycerol (DAG) synthesis, eventually generating monoacylglycerol, mainly 2-arachidonoyl glycerol (2-AG), via the OX-1R-Gq-PLCβ – DAGLα cascade, as documented in *in vitro* (Ammoun et al., 2006; Turunen et al., 2012; Imperatore et al., 2016), and *in vivo* studies (Ho et al., 2011). Some studies have already demonstrated that 2-AG can be metabolized to 2-arachidonoyl LPA (hereafter named 2-AGP) by a monoacylglycerol kinase (Kanoh et al., 1986; Shim et al., 1989). Many subtypes of LPAs and LPA-specific G-protein-coupled receptors (LPA1–6) are present in the brain (Choi and Chun, 2013). The LPA species 2-AGP, and its receptor LPA1 (or EDG2), hereafter referred to as LPA1R, are prevalent in both embryonic and adult mammalian brains (Nakane et al., 2002). LPAs have been demonstrated to promote the GSK3β-mediated microtubule depolymerization, neurite extension and axon differentiation by regulating tau phosphorylation under physiological and pathological conditions such as brain development and AD, respectively (Hao et al., 2020). Accordingly, several pieces of evidence indicate a regulatory role of LPA1R in promoting synaptic modifications in adult hippocampal neurons (Fujiwara et al., 2003; Jin Rhee et al., 2006; Castilla-Ortega et al., 2011; Roza et al., 2019). Although a significant increment of OX-A or 2-AG levels has been documented in the plasma of AD patients (Koppel et al., 2009; Altamura et al., 2015), the possible 2-AG-derived production of 2-AGP and the phosphorylation of Tau as a downstream effect of 2-AGP signaling at LPA1R, have not been yet addressed.

The present study is aimed to explore the role of the specific endocannabinoid 2-AG-derived lysophosphatidic acid 2-AGP in the Tau-mediated microtubule assembly and maintenance of axonal homeostasis, and its impairment in AD. For this purpose, we investigated the effects of OX-A and 2-AGP on the phosphorylation of threonine 231 of Tau protein (pT231-Tau), which has been identified as a sensitive and specific early biomarker for AD diagnosis (Kiđemet-Piskač et al., 2018; Suárez-Calvet et al., 2020; Gonzalez et al., 2022). Here, we analyzed plasma and CSF levels of 2-AGP and the correlation between OX-A and 2-AG plasma levels in patients with AD compared to age-matched healthy controls (HC). Furthermore, by confocal microscopy, LC-MS spectrometry, biochemical, molecular and patch clamp recording techniques, we investigated the molecular pathway linking OX-A, 2-AG and 2-AGP to the phosphorylation of threonine 231 of the Tau protein in hippocampal neurons and mouse brain, and its impact on the glutamatergic transmission in the hippocampus, which represents the brain neuronal substrate of most cognitive processes.

## Results

### Orexin-A, 2-arachidonoylglycerol and 2-arachidonoylglycerol-derived 2-arachidonoyl lysophosphatidic acid levels are increased in Alzheimer’s disease patients

**FIGURE 1:**
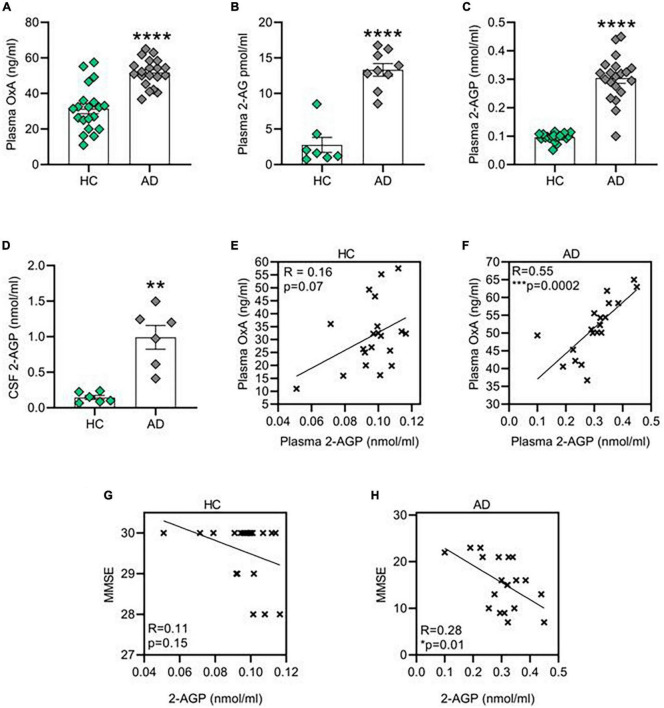
Orexin-A, 2-AG and 2-AG derived 2-AGP are increased in patient affected by Alzheimer’s disease (AD). **(A)** Bar graph with mean ± SEM of OX-A levels in the plasma of healthy control and AD patients. HC (31.42 ± 2.8 ng/ml) vs. AD (51.71 ± 1.7 ng/ml), ****p < 0.0001 Unpaired two-tailed T test; *n* = 20 HC and 20 AD. **(B)** Bar graph with mean ± SEM of OX-A levels in the plasma of healthy HC and AD patients. HC (2.76 ± 1 pmol/ml) vs. AD (13.31 ± 0.8 pmol/ml), ***p < 0.001 two-tailed Mann–Whitney Test, *U* = 0, *n* = 7 HC and 9 AD. **(C)** Bar graph with mean ± SEM of 2-AGP levels in the plasma of healthy HC and AD patients. HC (0.09 ± 0.003 nmol/ml) vs. AD (0.3 ± 0.01 nmol/ml), ****p < 0.0001 two-tailed Mann–Whitney Test, *U* = 8.5, *n* = 20 control and *n* = 20 AD. **(D)** Bar graph with mean ± SEM of the 2-AGP levels in the cerebrospinal fluid of HC and AD patients. HC (0.14 ± 0.02 nmol/ml) vs. AD (0.99 ± 0.16 nmol/ml), **p < 0.0001 Unpaired two-tailed T-test, *n* = 6 control and 6 AD. **(E)** Correlation between the OX-A and 2-AGP levels in the plasma of HC patients. Pearson’s correlation coefficient, *p* (two tailed) = 0.07. **(F)** Correlation between the OX-A and 2-AGP levels in plasma of AD patients. Pearson’s correlation coefficient, ***p (two tailed) < 0.001**. (G,H)** Correlation between 2-AGP levels in the plasma of HC **(G)** or AD **(H)** subjects with the respective score obtained from each mini-mental state examination (MMSE). Pearson’s correlation coefficient *p* (two tailed) = 0.15 in HC and *p (two tailed) = 0.01 in AD patients.

**FIGURE 2:**
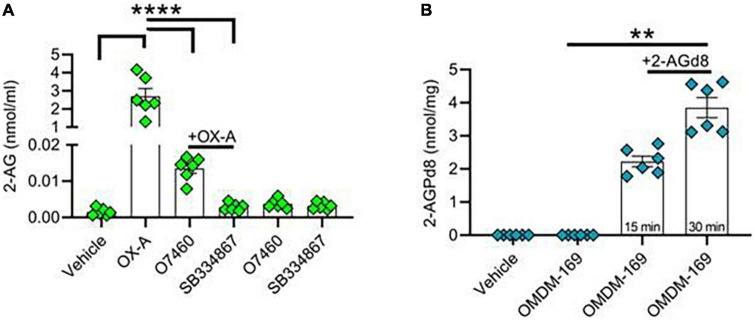
Orexin-A raises 2-AG and 2-AG-derived 2-AGP levels in hippocampal neurons. **(A)** Bar graph with mean ± SEM. of 2-AG levels in hippocampal primary neurons in different experimental condition: vehicle, OX-A (300 nM), OX-A (300 nM), + O7460 (10 μM), OX-A (300 nM), + SB334867 (10 μM), O7460 (10 μM), SB334867 (10 μM). 2-AG levels: vehicle (0.001 ± 0.0004 nmol/ml), OX-A (2.6 ± 0.4 nmol/ml), OX-A + O7460 (0.01 ± 0.001), OX-A + SB334867 (0.002 ± 0.0004 nmol/ml), O7460 (0.003 ± 0.0005 nmol/ml), SB334867 (0.003 ± 0.0003 nmol/ml), *n* = 6 wells per experimental group, one-way ANOVA with Bonferroni *post hoc* test, *F* = 6.3. **(B)** Bar graph with mean ± SEM. of 2-AGPd8 levels in hippocampal primary neurons in different experimental conditions: Vehicle, OMDM-169 (10μM), 2-AGd8 (50μM) + OMDM-169 (10μM) for 15 and 30 min of incubation. 2-AGPd8 levels: vehicle and OMDM-169 (undetectable), 15 min 2-AGd8 + OMDM-169 (2.22 + 0.15 nmol/mg), 30 min 2-AGd8 + OMDM-169 (3.8 ± 0.3 nmol/mg), Kruskal–Wallis test and Dunn’s *post-hoc* test, Kruskal–Wallis statistic 22.20, **p < 0.01.

Here we report a parallel raise of OX-A (Figure 1A), 2-AG (Figure 1B) and 2-AGP (Figure 1C) concentrations in the plasma collected from patients in an AD cohort (Supplementary Table 1) as compared to the gender- and age-matched healthy controls (HC) (^****^p < 0.0001). Furthermore, an enhancement of 2-AGP levels was detected in the CSF collected from a different set of AD patients (Figure 1D). Notably, a significant positive correlation between OX-A and 2-AGP was observed in the plasma of AD patients (^***^p < 0.001), but not in the plasma from HC subjects (*p* = 0.07) (Figures 1E,F). Although no significant correlation between 2-AG plasmatic levels and the mini-mental examination (MME) scores was found in either HC (*R* = 0; *p* = 0.9) or AD subjects (*p* = 0.5) (Supplementary Figure 1), a negative correlation of 2-AGP levels with the MMSE scores was found in AD subjects (*p < 0.05) but not in the healthy controls (*p* = 0.15) (Figures 1G,H).

### Orexin-A triggers the production of 2-arachidonoylglycerol, a precursor of 2-arachidonoyl lysophosphatidic acid biosynthesis

In an *in vitro* model of mouse hippocampal primary neurons, we first demonstrated an OX-A-induced 2-AG production after 45 min of cell incubation with 300 nM OX-A (Figure 2A). These levels were lowered by OX-A incubation in the presence of O7460, a DAGLα inhibitor, or SB334867, an OX-1R antagonist (Figure 2A). Of note, 15 min and 30 min incubation of hippocampal neurons in the presence of deuterated 2-AG (2-AGd8) and OMDM-169, a potent and selective inhibitor of the monoacylglycerol lipase (MAGL) (Bisogno et al., 2009), the main enzyme responsible for 2-AG degradation, led to a significant production of 2-AGPd8, as assessed by LC-MS spectrometry (Figure 2B).

### Orexin-A enhances pT231-tau phosphorylation in hippocampal primary neurons

**FIGURE 3:**
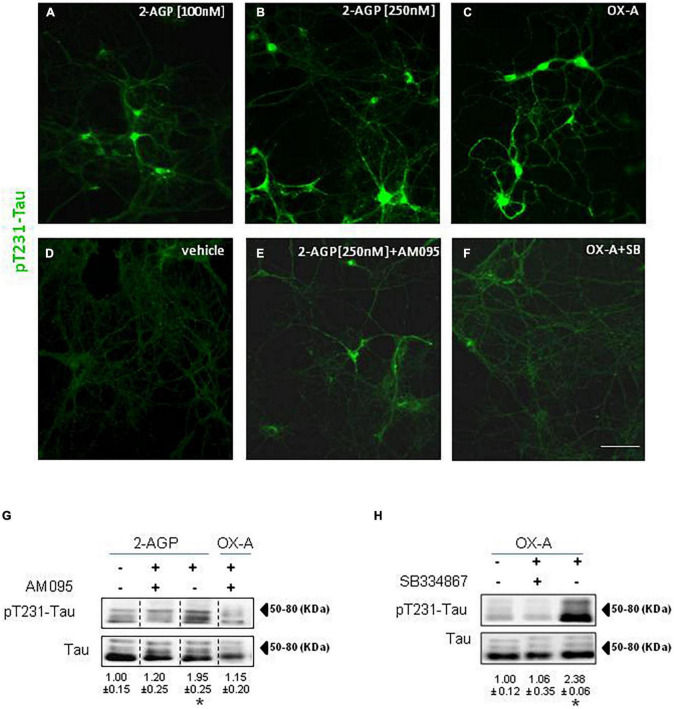
Phosphorylation of Tau is induced by 2-AGP via OX-A and destabilizes microtubules of primary neuronal culture. **(A–F)** Representative images of pT231-Tau immunoreactivity in the primary neurons from mouse hippocampus under different experimental conditions show the highest expression in the soma and neural processes of cells treated with 2-AGP and OX-A that is blunted by incubation with the respective receptor antagonist AM095 or SB334867; Scale bar = 100 μm. **(G)** Representative immunoblots of pT231-Tau/Tau ratios showing the effect of the 2-AGP or OX-A, with or without incubation with the LPA1R antagonist AM095 (10μM) of primary hippocampal neurons. Lanes from blots spliced together in a composite image are separated by a dotted black vertical line. Data are means ± SEM (*n* = 3 mice/group). Optical density (OD) values are expressed as the ratio between the OD values of phosphorylated and total protein. Data represent means ± SEM, statistical analysis was performed by ANOVA followed by Tukey’s test. *p < 0.01. **(H)** Representative immunoblots of pT231-Tau/Tau ratios showing the effect of OX-A, with or without incubation with the OX-1R receptor antagonist SB334867 (10 μM). Data are means ± SEM (*n* = 3 mice/group). Optical density (OD) values are expressed as ratio between the OD value of phosphorylated and total protein. Data represent means ± SEM, statistical analysis was performed by ANOVA followed by Tukey’s test; *p < 0.01.

We treated hippocampal primary neurons with 2-AGP or OX-A to evaluate the pT231-Tau immunoreactivity. We observed a concentration-dependent increment of pT231-Tau immunolabeling in the cytoskeleton of hippocampal primary neurons treated with 100 nM 2-AGP (Figure 3A) or 250 nM 2-AGP (Figure 3B) with respect to the untreated cells (Figure 3D). The 2-AGP-mediated expression of pT231-Tau immunoreactivity was prevented by incubation with AM095, a selective LPA1R antagonist (Figure 3E). In line with OX-A-driven 2-AGP production via the putative OX1R-Gq-PLCβ – DAGLα-2-AG pathway, incubation of the hippocampal primary neurons with 300 nM OX-A induced an increment of the pT231-Tau immunoreactivity in comparison to the untreated cells (Figure 3C) and in a manner prevented by the OX1-R inhibitor SB334867 (Figure 3F). Furthermore, we quantified OX-A- or 2-AGP-induced pT231-Tau production by western blot analysis in hippocampal primary neurons treated with each of these molecules. We found a significant increment of pT231-Tau immunosignal induced by 2-AGP or OXA (Figures 3G,H) and prevented by AM095 (Figure 3G) or SB334867 treatment (Figure 3H), respectively.

### 2-Arachidonoyl lysophosphatidic acid induces pT231-Tau production and impairment of glutamatergic transmission in the CA1 region of the mouse hippocampus

**FIGURE 4:**
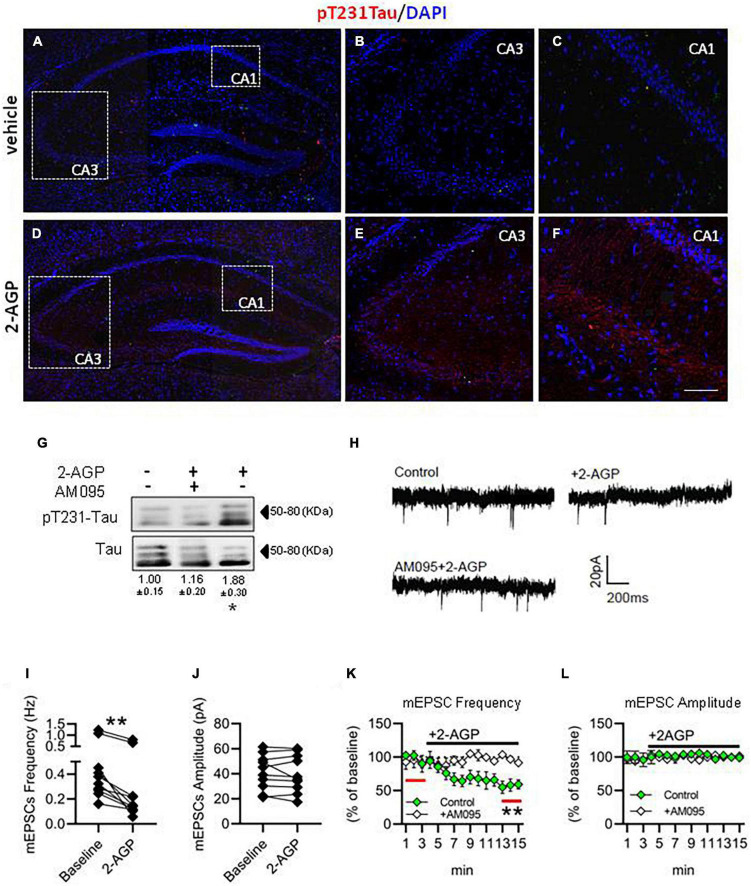
2-AGP triggers pT231-Tau phosphorylation and impairs glutamatergic transmission in the CA1 area of the hippocampus. **(A–F)** Representative images of pT231-Tau immunoreactivity in the hippocampus showing the highest expression in the CA3-CA1 area of 2-AGP-injected mice. Panels **(B,C,E,F)** are high magnification of the respective area selected by the dotted box in the panels **(A,D)**. Scale bar: 150 μm in **(A,D)** and 100 μm in **(B,C,E,F)**. **(G)** Representative immunoblots of pT231-Tau/Tau ratios showing the effect of 2-AGP (10 mg/kg, 1 h) or AM095 treatment (10 mg/kg), injected, 1 h before 2-AGP. Optical density (OD) values are expressed as the ratio between the OD values of phosphorylated and total protein. Data represent means ± SEM (*n* = 3 mice/group), *p < 0.05, statistical analysis was performed by ANOVA followed by Tukey’s test. **(H)** Representative recordings traces of mEPSCs in CA1 pyramidal neurons in control, 2AGP and AM095 + 2-AGP. **(I)** Graph of the mEPSC frequency before (3 min) (0.48 ± 0.11 Hz) and after treatment with 250 nM 2-AGP (3 min) (0.25 ± 0.08 Hz), Wilcoxon matched-pairs signed rank test. **p < 0.01, *n* = 10 neurons in 4 mice **(J)** Graph of the mEPSC amplitude before (41.28 ± 4.4 pA) and after 2-AGP (40.10 ± 4.7 pA), *n* = 10 neurons in 4 mice. **(K)** mEPSC frequency (% of 3 min of baseline). Green: effect of the 2-AGP treatment in CA1 pyramidal neurons, white: effect of the 2-AGP treatment in pyramidal neurons pre-treated with AM095 (10μM), statistical analysis was performed by two-tailed one sample t-test, **p < 0.05; red lines represent the part of compared graph. **(L)** mEPSC amplitude (% of the baseline). Green: effect of the 2-AGP treatment in CA1 pyramidal neurons, white: effect of the 2-AGP treatment in pyramidal neurons pre-treated with AM095.

To understand if 2-AGP could trigger pT231-Tau accumulation in the hippocampus *in vivo*, we injected C57Bl/6J mice with 2-AGP (10 mg/kg, 1 h) and revealed pT231-Tau immunoreactivity (Figure 4). We observed an increment of pT231-Tau immunolabeling in the CA3 and CA1 areas (Figures 4D–F) in a manner prevented by injection of AM095 (10 mg/kg, 1 h before 2-AGP) (Supplementary Figure 2). Furthermore, quantification of 2-AGP-induced pT231-Tau immunoreactivity was performed by western blot analysis of the hippocampal region of mice injected with 2-AGP (10 mg/kg, 1 h) or 2-AGP + AM095 (10 mg/kg, 1 h before 2-AGP) (Figure 4G).

To investigate the effect of 2-AGP on excitatory synaptic transmission, we measured the frequency and amplitude of the miniature excitatory postsynaptic current (mEPSCs) under control conditions (3 min) and upon bath application of 250 nM 2-AGP by voltage-clamp recordings in CA1 pyramidal neurons. We observed a reduction of the mEPSCs frequency (Figures 4H,I,K) (^**^p < 0.05), while amplitude was unchanged (Figures 4H,J,L). The pretreatment of hippocampal slices with AM095 prevented the effect of 2-AGP on the mEPSCs frequency (Figures 4K,L).

## Discussion

In the present study, we reported that: (i) OX-A, 2-AG and 2-AGP levels are enhanced in the plasma of AD patients compared to age-matched healthy controls; (ii) 2-AGP levels are enhanced in the CSF of AD patients; (iii) a positive correlation exists between OX-A and 2-AGP plasma levels of AD patients but not of healthy controls; (iv) pT231-Tau levels are elevated in hippocampal primary neurons and in the CA1, CA3 subfields of the mouse hippocampus following administration of 2-AGP or OX-A, in a manner abolished by previous treatment with OX1R, LPA1R and/or DAGLα blockers; (v) 2-AGP impairs glutamatergic neurotransmission in the mouse CA1 hippocampal subfield; and (vi) plasmatic 2-AGP levels in AD patients correlate negatively with a measure of cognitive function.

Regular sleep-wake behavior plays a positive function in brain homeostasis by preserving synaptic plasticity and brain function from neurodegenerative injuries (Tononi and Cirelli, 2006; Mander et al., 2016). Elevation of CSF orexin-A levels has been described in AD patients (Osorio et al., 2016; Gabelle et al., 2017; Shimizu et al., 2020). In line with this evidence, epidemiological data report sleep disturbances in about half of AD patients (Liguori et al., 2021).

Extensive data support the existence of OX-A-induced endocannabinoid 2-AG production (Ammoun et al., 2006; Turunen et al., 2012; Imperatore et al., 2016; Berrendero et al., 2018). A study investigating 2-AG concentrations in the plasma of AD patients showed a significant increment in comparison to age-matched controls (Altamura et al., 2015). Here, we report for the first time that elevation of both OX-A and 2-AG occurs in the plasma of AD patients.

Several investigators have demonstrated that 2-AG can be metabolized to 2-arachidonoyl LPA (herein named 2-AGP), a selective ligand of LPA1R (Kanoh et al., 1986; Shim et al., 1989; Nakane et al., 2002), which modulates synaptic plasticity, Tau phosphorylation and cognitive function (Castilla-Ortega et al., 2011; Roza et al., 2019). Our data report elevation of 2-AGP in CSF and plasma of AD patients in agreement with recent findings that indicate the failure of cognitive improvement after JZL-195 pharmacological inhibition of MAGL and FAAH activities in mouse model of AD (Bajaj et al., 2022).

Conversely, activation of CB1 receptor by enhancing endocannabinoid tone has been shown to inhibit Tau phosphorylation in animal models of AD and, in some cases, to ameliorate cognitive functions (Hashem et al., 2021).

In this study, by adding deuterated 2-AG to the culture medium of primary hippocampal neurons, we demonstrated the production of deuterated 2-AGP. Additionally, we provided unprecedented evidence of OX-A-induced, and DAGLα-mediated, 2-AGP production in these neurons. Thus, although 2-AG levels, *per se*, do not have utility as diagnostic biomarkers for AD since a robust correlation with cognitive performance is missing (Cristino et al., 2020), in this study we first documented an association between cognitive function and the levels of 2-AG-derived 2-AGP in the plasma or CSF of AD patients.

CB1 antagonism reduces sleep time and causes sleep fragmentation (Martin et al., 2022), which in turn elevates CSF orexin signaling (Olsson et al., 2018) and 2-AG plasma levels in humans (Cedernaes et al., 2016; Hanlon et al., 2016), thus supporting a potential detrimental function of OX-A and 2-AGP in chronically sleep-deprived individuals. On the other hand, associations between OX-A and AD have been extensively investigated and several studies suggest that over-activity of orexinergic system might be associated with AD pathology (Liguori et al., 2021). In older non-demented people an association between CSF OX-A levels and poor sleep quality has been described (Osorio et al., 2016). CSF OX-A levels were found to directly correlate with tau levels in healthy control subjects (Osorio et al., 2016; Gabelle et al., 2017; Shimizu et al., 2020) and in AD patients (Deuschle et al., 2014).

A multicenter longitudinal cohort study refers to the quantification of pT231-Tau levels as a cost-effective and accurate biomarker in assessing cognitive decline in humans (Gonzalez et al., 2022). Moreover, p-tau231 concentration *per se* has also been correlated with the progression of AD (Kiđemet-Piskač et al., 2018), being elevated in subjects with MCI in preclinical AD and inversely correlated with their MMSE score (Buerger et al., 2002b; Chatterjee et al., 2022). Accordingly, our data support that OX-A-dependent production of 2-AG and 2-AGP is associated with impairment of cognitive function since a positive correlation between MMSE and 2-AGP levels occurs in the plasma of AD patients. Of note, this is the first study where OX-A plasma levels have been quantified in AD and healthy subjects in correlation with 2-AGP levels.

In summary, we hypothesize that the daytime sleepiness and wake fragmentation occurring in AD patients, with moderate-severe stages of the disease, could be ascribed to the enhancement of plasma and/or CSF OX-A and 2-AG levels. This unbalance may trigger a vicious circle underlying further sleep disturbance and subsequent impairment of β-amyloid removal (Altamura et al., 2015; Osorio et al., 2016; Gabelle et al., 2017; Shimizu et al., 2020), by favoring Tau phosphorylation, neurodegeneration and alteration of synaptic plasticity and glutamatergic signaling in the hippocampus. LPA receptors are widely distributed in the brain and LPA1R has a regulatory role in promoting synaptic modifications in adult hippocampal neurons by controlling Tau phosphorylation (Fujiwara et al., 2003; Jin Rhee et al., 2006; Castilla-Ortega et al., 2011; Roza et al., 2019). Evidence exists concerning Tau pathology causing dysfunction of synapses and irreparable synaptic loss associated with cognitive impairment in AD patients (Kiđemet-Piskač et al., 2018). Since pT231-Tau is a well-recognized specific early CSF marker of AD (Hampel et al., 2001; Buerger et al., 2002a,b), here we focused on the molecular pathway linking OX-A to pT231-Tau production by revealing a 2-AGP-mediated pT231-Tau phosphorylation and its detrimental effect on the hippocampal glutamatergic network underlying memory and cognitive function, in agreement with previously reported findings (Garcia-Morales et al., 2015; Roza et al., 2019).

Despite the main limitation of this study concerning the small number of plasma donors (*n* = 20) and the even smaller number of CSF donors (*n* = 6), mainly because of a ethical problem in collecting CSF from normal subjects, it is important to highlight that all the participants were selected among those with sleep disturbances, day/night sleep fragmentation or general insomnia, as referred by themselves or their caregivers. In view of the biochemical, pharmacological, morphological and electrophysiological evidence here reported in hippocampal primary neurons and in the hippocampus of mice, these results suggest that orexin antagonism by conventional pharmacological insomnia treatments may be beneficial in subjects with mild-to-moderate age-related cognitive decline associated with sleep disturbances to delay or limit neurodegenerative injuries. Although several anti-AD molecules are under the drug development pipeline for human therapy, the possible use of LPA1R antagonists could also be considered in conjunction with the beneficial effect on synaptic plasticity and cognition, reported in preclinical and human studies, with inhibitors of 2-AG metabolism (Zhang et al., 2014; Berry et al., 2020).

## Materials and methods

### Subject population

The study was approved by the Regional Health Authority of the University of Molise. Written informed consent was obtained from subjects or caregivers, who were completely informed about the procedures. The ethical principles of the Declaration of Helsinki, and the national and international guidelines for human research were followed. Forty male participants, 20 healthy subjects (all males with mean age ± SD of 69.26 ± 26.21) and 20 with AD (all males with mean age ± SD of 76.70 ± 8.12), were recruited from the Centre for Research and Training in Medicine for Aging (CeRMA), University of Molise (Italy). Patients with Alzheimer’s clinical syndrome were diagnosed according to National Institute on Aging/Alzheimer’s Association (NIA–AA) criteria and fulfilled the criteria for the “probable AD with documented decline” category (McKhann et al., 2011). They presented Mini-Mental State Examination (MMSE) score < 24 and Clinical Dementia Rating (CDR) score > 0.5 (Folstein et al., 1975). The patients under treatment with cerebro-active drugs underwent a washout period of at least 14 days before the assessment.

### Human sample collection and preparation

Venous blood was collected with standard clinical procedures between 7:30 and 8:00 am after overnight fasting. For the collection we used vacutainer plasma tubes (Becton & Dickinson, Milan, Italy); samples were coagulated at room temperature for 10 min, and then a 10-min centrifugation at 3,000 g was applied. Supernatants were frozen in liquid nitrogen and stored at –80°C until measurements. Plasma was used to analyze OX-A and 2-AGP levels.

### ELISA for orexin-A levels

OX-A levels from patient plasma were measured with ELISA KIT (Phoenix Pharmaceuticals Inc, Burlingame, CA, USA) according to the manufacturer’s instructions. Triplicate samples were assayed, and OX-A levels were determined against a known standard. OX-A levels were measured using an ultra-sensitive Fluorescent EIA Kit (Phoenix Pharmaceuticals Inc, Burlingame, CA, USA) following the manufacturer’s instructions. Before measurement, plasma OX-A was extracted using Sep-Pak C18 columns (Waters, Milford, MA). The sample, applied to the column, was eluted slowly with 80% acetonitrile. The samples were evaporated, and the dry residue was dissolved in water and used for Fluorescent EIA Kit.

### Cell culture and immunocytochemistry

Primary cultures of mouse cortical neurons, derived from neonatal or 1-day-old C57BL/6 (Charles River) mice were carried out as described (Palomba et al., 2015). Briefly, the cerebral cortex was quickly separated and mechanically dispersed in Ca^2+^- and Mg^2+^-free buffered Hanks’ balanced salt solution. Then, tissues were dissociated by both enzymatic (0.125% trypsin solution, 37°C for 20 min) and mechanic procedures. The cells were inoculated at a density of 2 × 10^4^ cells/cm^2^ on polylysine-coated coverslips and grown at 37°C in Neurobasal medium supplemented with 2% B27, 0.5 mM L-glutamine, penicillin (50 U/mL), and streptomycin (50 μg/mL), gassed with an atmosphere of 95% air and 5% CO_2_. Cells were used between 6 and 8 d *in vitro*. More than 80% of primary cultured cells were positive for neuronal marker NeuN antibodies, determined by immunocytochemistry (data not shown). Cell cultures were treated with 2-AGP (100 and 250 nM for 45 min) with or without AM095 (250 nM), or OX-A (300 nM for 45 min) or OX-A + SB334867 (10 μM for 15 min). After treatments, the cells were washed three times with PBS (#14190094 Thermo Fisher) and, finally, fixed for 20 min with 4% (wt/vol) paraformaldehyde (#P6148, Sigma)/0.1M phosphate buffer (PB) pH 7.4. These preparations were rinsed with PBS and immunostained by incubation of cells with 1:1,000 mouse anti-pThr231-Tau (#sc-32276, Santa Cruz 544 Biotechnology), after that were washed with PB and immunofluorescence revealed by specific Alexa secondary donkey anti-IgGs (Invitrogen, ThermoFisher Scientific, France) Alexa- 488 donkey anti-mouse. The cell specimens were mounted with Prolong Gold (Invitrogen), and coverslipped with Aquatex mounting medium (Merck, Darmstadt, Germany). Stained cells were analyzed with a Leica DMI6000 fluorescence microscope equipped with a Leica K5 cooled digital CCD camera (Leica Microsystems).

### Animals

The study has been performed according to the ARRIVE Guidelines to improve the reporting of bioscience research using laboratory animals. Experiments were performed following the European Union animal welfare guidelines [European Communities Council Directive of September 22, 2010 (2010/63/EU)] and the Italian Decree n.26/2014, authorization n. 152/2020-PR and 589/2018. All experiments were carried out on 8-week-old male wild-type C57BL/6J purchased from Charles River (Calco, Italy). All the mice were housed in controlled temperature (20-23°C) and humidity conditions (55 ± 5%), and fed *ad libitum*. All animals were used in scientific experiments for the first time and were not previously exposed to any pharmacological treatments. Mice were injected intraperitoneally with vehicle (saline), 2-AGP (Avanti Polar Lipids, 10 mg/kg, 1 h), AM095 (MedChemExpress, 10 mg/kg, 1 h before 2-AGP injection).

### Immunohistochemistry

The animals were deeply anesthetized and transcardially perfused with physiological saline at RT. Following saline, the animals were perfused with 4% paraformaldehyde in 0.1M phosphate buffer (PB), pH 7.4. The brains were cut with a Leica CM3050S cryostat into 10-μm-thick serial sections in the coronal plane, collected in alternate series, and processed for immunofluorescence. Anatomic comparable sections of the hippocampus were processed for immunofluorescences after incubation for 1 h at room temperature in PB containing 0.3% Triton and 5% donkey plasma (blocking buffer). Sections were then incubated overnight at 4°C with the primary antibodies diluted in donkey plasma. The following primary antibodies were used: 1:1,000 mouse anti-pThr231 Tau (#sc-32276, Santa Cruz 544 Biotechnology). After incubation with primary antibodies, the sections were washed with PB and immunofluorescence was revealed by specific Alexa secondary donkey anti-IgGs (Invitrogen, ThermoFisher Scientific, France) or Alexa-594 donkey anti-mouse (A21203, 1:50). Sections were counterstained with DAPI (Sigma-Aldrich) to detect nuclei, mounted and coverslipped with Aquatex mounting medium (Merck, Darmstadt, Germany). Controls of specificity of immunolabeling were performed by omission of primary and secondary antibodies or by preabsorption of primary antibodies with the respective blocking peptides. The immunostained sections were observed with a Nikon Eclipse Ti2 microscope (Nikon, Florence, Italy) equipped with an x-y-z motorized stage, a digital camera DS-Qi2 (Nikon, Florence, Italy), and the acquisition and Image analysis software NIS-Elements C (Nikon, Florence, Italy). Digital images were acquired using the ×20-×40 objectives. A serial Z-stacks of images were collected throughout the area of interest (*n* ≤ 10 planes with an increment varying 0.5–1 μm). Images were deconvolved using the imaging deconvolution software by application of *n* = 10 iterations. Serial Z plane images were collapsed into a single maximum projection image. Micrographs were saved in TIFF format and adjusted for light and contrast before being assembled on plates using Adobe Photoshop 6.01 (Adobe Systems, San Jose, CA).

### Lipid extraction and 2-arachidonoylglycerol/2-arachidonoyl lysophosphatidic acid measurement from plasma and cell culture

Hippocampal primary neurons were treated with OX-A (Tocris, 300 nM for 30 min) in the absence or presence of SB334867 (Tocris, 10 μM for 45 min *per se* or 15 min before OX-A exposure) or O7460 (Cayman Chemical, 10 μM for 45 min *per se* or 15 min before OX-A exposure) or OMDM-169 (Bisogno et al., 2009) (10μM for 45 min *per se* or 15 min before OX-A exposure). Each sample contained 0.5 × 10^5^ cells/mL. After treatment, cells and supernatant were collected, homogenized, and analyzed. Samples were pooled and homogenized in 5 vol chloroform/methanol/Tris HCl 50 mM (2:1:1 by volume) containing 50 pmol of d5-2- arachidonoylglycerol (d5-2-AG) as internal standards. Homogenates were centrifuged at 13,000 × *g* for 16 min (4°C), and the aqueous phase plus debris were collected and four times extracted with 1 vol chloroform. The lipid-containing organic phases were dried and pre-purified by open-bed chromatography on silica columns eluted with increasing concentrations of methanol in chloroform. Fractions for 2-AG or 2-AGP measurement were obtained by eluting the columns with 9:1 (by volume) chloroform/methanol and then analyzed by liquid chromatography-atmospheric pressure chemical ionization-mass spectrometry (LC-APCI-MS). LC-APCI-MS analyses were carried out in the selected ion monitoring mode, using m/z values of 384.35 and 379.35 (molecular ions + 1 for deuterated and undeuterated 2-AG). 2-AG/2AGP levels were normalized per mL of cell + medium or ml of plasma in the case of the patient blood samples.

### Western blot analysis from mice sample and cell culture

Each animal, previously anesthetized with isoflurane for 5 min, was decapitated and the hippocampus was quickly removed and washed twice in cold PBS (without Ca2 + and Mg2+, pH 7.4) and homogenized in lysis solution (1 × TNE Buffer, 1% (v/v); TritonX-100, plus 1% protease inhibitor cocktail) at pH 7.4. Lysates were kept in an orbital shaker incubator at 220 × g, at 4°C for 30 min, and then centrifuged for 15 min at 13,000 × *g* at 4°C. The supernatants were transferred to clear tubes and quantified by the DC Protein Assay (cat. 5000111, Bio-Rad, Milan, Italy). Subsequently, the samples (60 μg of total protein) were boiled for 5 min in Laemmli SDS loading buffer and loaded on 8–10% SDS-polyacrylamide gel electrophoresis and then transferred to a PVDF membrane.

Cell samples were homogenized in 1xTNE buffer (50 mM Tris pH 7.4, 150 mM NaCl, 1 mM EDTA) containing 10% Triton X-100, protease, and phosphatase inhibitor mixtures (Sigma Aldrich) for obtaining protein extracts. Protein concentrations were analyzed using Lowry protein assay (Bio-Rad Laboratories) to allow the loading of the same amount of proteins (20 μg) Proteins were separated in an SDS-polyacrylamide gel (4–20%) by electrophoresis and transferred to PDVF membranes, which were then blocked for 1 h with 5% skim milk powder dissolved in 1xTBST (20 mM Tris, 137 mM NaCl, 0.1% Tween-20).

After blocking, the membranes were incubated overnight at 4°C with the following primary antibodies: mouse anti-Tau (1:900, #4019S, Cell Signaling); rabbit anti-pThr231 Tau (1:1000, #44746G, Invitrogen); mouse anti-PSD95 (1:2000, #124011, Synaptic system), and mouse monoclonal anti-βactine (1:4000, #A1978, Sigma Aldrich), which was used to check for equal protein loading. After washing in TBST, the membranes were incubated for 1 h at room temperature with HRP conjugated goat anti-rabbit secondary antibody (1:4000, #1706515 Biorad) or goat anti-mouse (1:4000, #1706516, Biorad).

Immunoreactive bands were visualized using enhanced chemoluminescence (Clarity ECL, #1705061, Biorad) and then exposed to a ChemiDoc MP Imaging System (# 17001402, Biorad). Bands were quantified using ImageJ software (NIH, USA). When necessary, membranes were stripped after protein detection for 10 min at 55°C with a solution containing 62.5 mmol/l Tris-HCl, 100 mmol/l 2-mercaptoethanol, 582, and 2% SDS, blocked, and reblotted with another primary antibody.

### Electrophysiology

Acute hippocampal slices were prepared from 4 to 8 weeks-old C57BL/6J mice. Mice were anesthetized by a brief exposure to isoflurane, decapitated and the brain dissected in an ice-cold (2–5°C) cutting media containing (in mM): 87 NaCl, 25 NaHCO_3_, 2.5 KCl, 0.5 CaCl_2_, 7 MgCl_2_, 25 glucose, 75 sucrose and saturated with 95% O_2_ - 5% CO_2_. Hippocampal coronal slices of 350 μm were obtained using a Leica VT1000 S Vibrating. Slices were allowed to recover for 30–45 min at 35°C in a constantly 95% O_2_ - 5% CO_2_ gassed recording artificial cerebrospinal fluid (ACSF) solution containing (in mM): 125 NaCl, 25 NaHCO_3_, 25 glucose, 2.5 KCl, 1.25 NaH_2_PO_4_, 2 CaCl_2_, and 1 MgCl_2_. Following recovery, slices were kept at RT. K-gluconate intracellular solution was used containing in mM: 126 K-gluconate, 4 NaCl, 1 MgSO_4_, 0.02 CaCl_2_, 0.1 BAPTA, 15 glucose, 5 HEPES, 3 MgATP, and 0.1 NaGTP, pH 7.3, 290 mosmol/l. Patch-clamp experiments were performed with borosilicate pipettes, pulled with a Sutter P-1000 puller to a final resistance of 3–4 mΩ, using the Multiclamp 700B (Molecular Devices, Sunnyvale, CA). Data were acquired with a 2 kHz low-pass Bessel filter and digitized at 10 kHz with Clampex 11.1 and analyzed offline with Clampfit 11.2 (pClamp, Molecular Devices, Sunnyvale, CA). Whole-cell recordings were performed on visually identified CA1 pyramidal neurons (–70 mV). Miniature excitatory postsynaptic currents (mEPSCs) were recorded in the presence of TTX (0.3 μM), Bicuculline (15 μM), CGP 55845 (5 μM) (all from Tocris Bioscience, Ellisville, MO). To test the effect of 2-AGP on frequency and amplitude of mEPSCs, 3 min of baseline were recorded before the application of the 2-AGP (250 nM) in the perfused solution. The effect of 2-AGP was statistically analyzed after 10 min.

### Statistic

Data were expressed as mean ± SEM, unless otherwise indicated, and were analyzed statistically using GraphPad Prism 8 (GraphPad Software, USA). In all tests, *p* < 0.05 was considered significant. Data are presented as a bar graph and single values were plotted. The correlation analysis was performed using Pearson’s correlation coefficient (r). The Shapiro-Wilkins and Kolmogorov-Smirnov tests were first applied to confirm the normal distribution of the data. Two-tailed unpaired T-test and Mann-Whitney test were used when 2 groups of data were compared. The Unpaired T-test was used when the compared groups were normally distributed while Mann-Whitney was applied in case one or both groups were non-normally distributed. One-way ANOVA with Bonferroni *post hoc* (in case of data normally distributed) or Kruskal–Wallis with Dunn’s tests (in case of a non-normal distribution) was used.

## Data availability statement

The original contributions presented in this study are included in the article/Supplementary material, further inquiries can be directed to the corresponding authors.

## Ethics statement

The studies involving human participants were reviewed and approved by Centre for Research and Training in Medicine for Aging (CeRMA), University of Molise (Italy). The patients/participants provided their written informed consent to participate in this study. The animal study was reviewed and approved according to the ARRIVE Guidelines to improve the reporting of bioscience research using laboratory animals. This study was reviewed and approved by the committee of the Italian Ministry of Health, approval number 152/2020-PR and 589/2018. Experiments were performed following the European Union animal welfare guidelines [European Communities Council Directive of September 22, 2010 (2010/63/EU)] and the Italian Decree n.26/2014, authorization n.152/2020-PR and 589/2018.

## Author contributions

NF designed and performed electrophysiological experiments, prepared the figures and wrote a drafted version of the manuscript. AF-R performed the western blot and biochemical studies. BM performed immunohistochemical experiments. LP designed and performed biochemical and cellular analysis and prepared the figures. FP performed biochemical analysis by LC-MS mass spectrometry. PD supervised pharmacological experiments in *in vivo* experiments. AD categorized and followed the enrolled subjects. LC and VD wrote the final version of the manuscript. LC conceived and supervised the study. All the authors discussed the data, edited and approved the final version of the manuscript.
