# Supplementary material for: Positive association between plasmatic levels of orexin A and the endocannabinoid-derived 2-arachidonoyl lysophosphatidic acid in Alzheimer’s disease
Source: Front Aging Neurosci. 2022 Nov 16;14:1004002. doi: 10.3389/fnagi.2022.1004002 (PMC9710385; doi:10.3389/fnagi.2022.1004002)
Supplement: Supplementary file 1 [file Data_Sheet_1.pdf]

Supplementary Table.1

	HC	AD		
**Age**	69,35 ±26,41	76,70 ±8,12	69.94	<0.001
**Schooling**	11,20 ±4,53	9,47 ±4,81	27.53	<0.001
**MMSE**	29,46 ±1,27	23,40 ±2,12	469,68	<0.001
**BMI**	23,9 ±1,59	23,40 ±2,12	21,806	<0.001
			X^2	P
**Sex**				
Male	20 (100%)	20(100%)		
			0.200	0.000
Female	0 (0%)	0(0%)		
**Patient history**				
Smoke	1 (10%)	6(30%)	39,200	0,361
Dyslipidemia	2(20%)	2(10%)	36,450	0,303
Diabetes	2(20%)	2 (10%)	39,200	0,516
Hypertension	4 (40%)	10(50%)	1,300	0,399
AMI	2(20%)	0(0%)	51,200	0,138
Cerebrovascular events	0(0%)	0 (0%)	76,050	0,393
**Drugs**				
Antihypertensive agent	7(70%)	14(70%)	2,450	0,240
Lipid lowering	1(10%)	2(10%)	16,200	0,971
**Medicinal**				
**Products**				
Thyroid hormons	1(10%)	2(10%)	34,667	0,06
AchE-I	0(0%)	4(20%)	63,450	0,292
Benzodiazepines	1(10%)	2(10%)	54,450	0,245

Supplementary Table 1. Demographic and clinical characteristics of the study group.

HC, healthy controls, AD, Alzheimer's disease. MMSE, Mini Mental State Examination.

Supplementary Fig.1

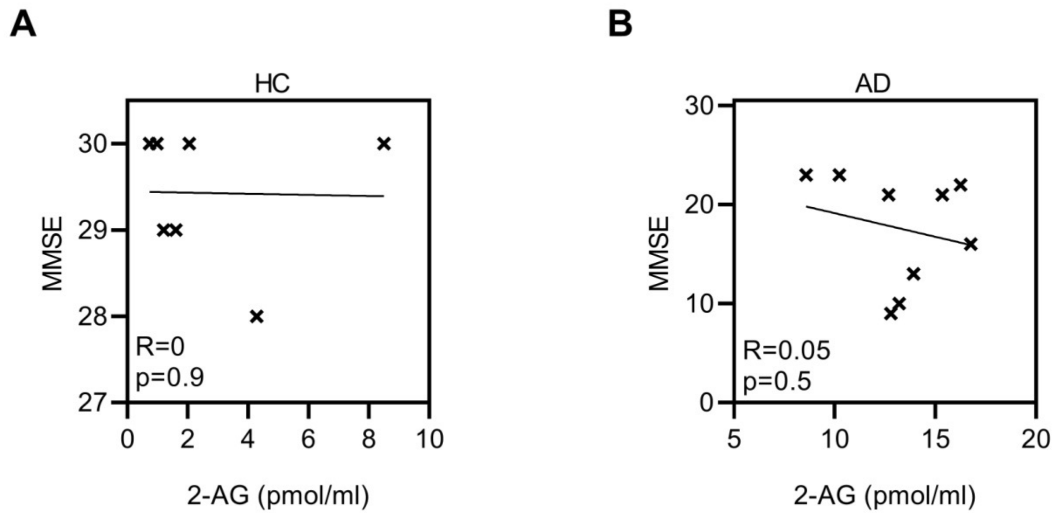

Supplementary Fig. 1. (**A**) Pearson's correlation between the 2-AG plasma levels and MMSE score in HC subjects, R=0; p (two tailed) = 0.15; (**B**) Pearson's correlation between the 2-AG plasma levels and MMSE score in AD subjects, R=0.05; p (two tailed) = 0.5.

Supplementary Fig.2

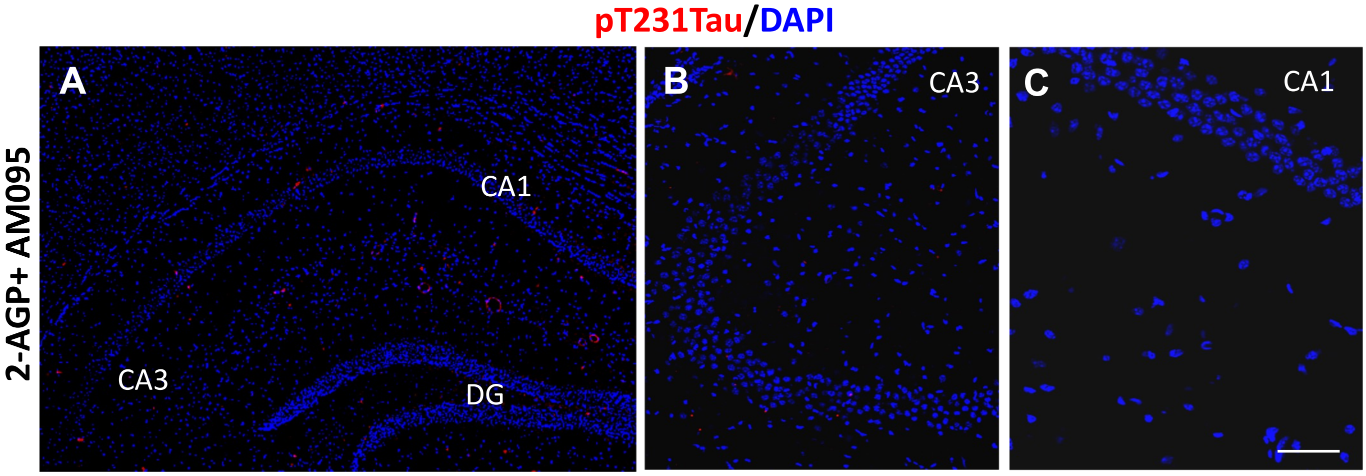

Supplementary Fig 2. Immunoreactivity of p-T231-tau in the hippocampus of mouse injected with the LPA1R antagonist AM095 before 2-AGP administration. Note the lowest pT231-tau expression.
